# Recent Progress in Electrochemical HbA1c Sensors: A Review

**DOI:** 10.3390/ma8031187

**Published:** 2015-03-17

**Authors:** Baozhen Wang, Jun-ichi Anzai

**Affiliations:** 1 Department of Nutrition and Food Hygiene, School of Public Health, Shandong University, 44 Wenhuaxi Road, Jinan 250012, Shandong, China; E-Mail: bzhenw@hotmail.com; 2 Graduate School of Pharmaceutical Sciences, Tohoku University, Aramaki, Aoba-ku, Sendai 980-8578, Japan

**Keywords:** electrochemical sensor, glucose sensor, HbA1c sensor, fructosyl valine sensor, blood glucose, diabetes mellitus

## Abstract

This article reviews recent progress made in the development of electrochemical glycated hemoglobin (HbA1c) sensors for the diagnosis and management of diabetes mellitus. Electrochemical HbA1c sensors are divided into two categories based on the detection protocol of the sensors. The first type of sensor directly detects HbA1c by binding HbA1c on the surface of an electrode through bio-affinity of antibody and boronic acids, followed by an appropriate mode of signal transduction. In the second type of sensor, HbA1c is indirectly determined by detecting a digestion product of HbA1c, fructosyl valine (FV). Thus, the former sensors rely on the selective binding of HbA1c to the surface of the electrodes followed by electrochemical signaling in amperometric, voltammetric, impedometric, or potentiometric mode. Redox active markers, such as ferrocene derivatives and ferricyanide/ferrocyanide ions, are often used for electrochemical signaling. For the latter sensors, HbA1c must be digested in advance by proteolytic enzymes to produce the FV fragment. FV is electrochemically detected through catalytic oxidation by fructosyl amine oxidase or by selective binding to imprinted polymers. The performance characteristics of HbA1c sensors are discussed in relation to their use in the diagnosis and control of diabetic mellitus.

## 1. Introduction

The development of electrochemical glucose sensors is a key subject in the field of biosensors [1,2,3]. Most glucose sensors consist of metal or carbon electrodes modified with glucose oxidase (GOx), which catalyzes the oxidation of glucose on the surface of the electrode. Recently, much attention has been devoted to the use of nano-materials for improving the performance characteristics of these sensors [4,5]. In addition, current research projects are focusing on the development of non-enzymatic glucose sensors [6,7]. Glucose sensors are widely used in diagnostic tests for diabetes mellitus in clinical laboratories and hospitals. One disadvantage of these tests is that the data obtained reflects the plasma glycemic status only at the moment of sample collection.

Blood glycated hemoglobin (HbA1c) is an indicator of diabetes mellitus. HbA1c forms when glucose binds to the *N*-terminal valine residue of the β-chains of hemoglobin [8]. The percentage of HbA1c in total hemoglobin reflects the average blood glucose level over the preceding 2–3 months because of the long life span of red blood cells in the circulation [9]. Therefore, HbA1c level is an important indicator that reflects the long-term glycemic state in the blood of diabetic patients. Normal HbA1c levels fall within the range 4%–6% [10]. A variety of techniques, including liquid chromatography [11,12], electrophoresis [13,14], affinity and ion-exchange chromatography [15,16], immunoassay [17,18], and spectrophotometry [19,20], are currently available for determining HbA1c levels. Although these techniques can provide accurate determination of HbA1c in clinical laboratories, they require expensive equipment and sometimes experienced operators. Developments in the detection of HbA1c using these techniques have recently been reviewed [21,22,23,24]. In contrast, electrochemical HbA1c sensors have been intensively studied with the aim of developing simple and inexpensive methods of detecting HbA1c levels in the blood. This review focuses on recent progress made in the development of electrochemical HbA1c sensors. These sensors are divided into two categories based on the modes of detection: direct detection of HbA1c by modified electrodes, or detection of the glycated amino acid fragment, fructosyl valine (FV), after proteolytic digestion of HbA1c. In the following section, we begin with an overview of HbA1c sensors using the direct detection mode.

## 2. Sensors for Direct Detection of HbA1c

Electrochemical HbA1c sensors are constructed by modifying the surface of electrodes with sugar-binding materials, including antibodies, binding proteins, and boronic acid. HbA1c sensors rely on different modes of signal transduction, such as amperometry/voltammetry, potentiometry, and impedometry. In this section, HbA1c sensors have been classified for convenience into the following categories: amperometric/voltammetric sensors, potentiometric sensors, impedometric sensors, and miscellaneous.

### 2.1. Amperometric/Voltammetric Sensors

**Figure 1:**
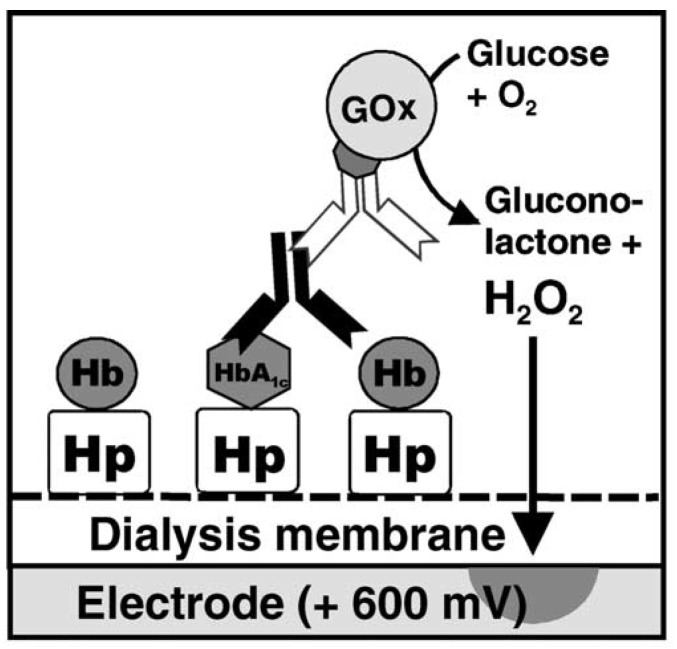
The response mechanism for amperometric HbA1c sensors based on haptoglobin (Hp), anti-HbA1c antibody, and GOx-labeled antibody. Reprinted with permission from Stöllner *et al.* [25].

The first amperometric HbA1c sensor was reported in 2002 by Stöllner and coworkers [25]. A haptoglobin-modified cellulose membrane was used to bind HbA1c onto the surface of an electrode. Haptoglobins are a group of serum proteins that bind hemoglobin released from erythrocytes in the blood stream [26]. Surface-confined HbA1c was then labeled with anti-HbA1c and GOx-labeled anti-immunoglobulin to generate amperometric signals (Figure 1). The output current of the sensor was acquired in the presence of 50 mM glucose as a specific substrate of GOx. This pioneering work demonstrated the potential of anti-HbA1c-modified electrodes as separation-free HbA1c sensors.

**Figure 2:**
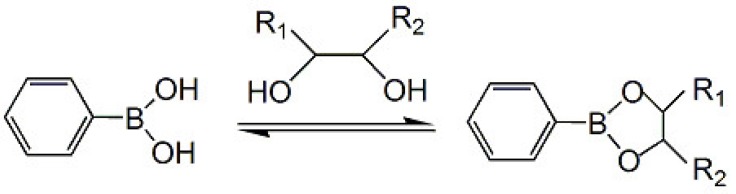
Binding equilibrium between phenylboronic acid and a diol.

Scheller and coworkers developed voltammetric HbA1c sensors using ferroceneboronic acid (FcBA) as a redox-active affinity label for HbA1c [27,28,29]. Boronic acid derivatives are known to form stable adducts with 1,2- and 1,3-diol compounds, including sugars, via covalent ester bonds (Figure 2) [30], and are widely used to construct non-enzymatic sensors for detecting sugars [31,32], catechins [33], lipopolysaccharides [34], steroids [35], phenols [36], nucleosides [37], lactate [38], and salicylate [39]. Incubation of zirconium dioxide nanoparticle-modified electrodes in sample solution composed of HbA1c and unmodified Hb followed by labeling with FcBA resulted in an FcBA-confined surface, which exhibited well-defined redox peaks at 0.3 V (*vs*. Ag/AgCl) in cyclic voltammetry. The magnitude of the redox peaks depended on HbA1c levels in the range 6.8%–14% [27]. In another protocol based on deoxycholic acid-modified electrodes, reusable sensors for HbA1c were developed. The HbA1c sensors could be used to determine 0%–20% HbA1c more than 30 times repeatedly after pepsin digestion of surface-deposited HbA1c [28].

Yoon and coworkers developed HbA1c sensors based on gold electrodes modified with poly(amidoamine) dendrimer bearing phenylboronic acid (PBA) [40] and a self-assembled PBA monolayer [41]. HbA1c was confined to the surface of the PBA-modified electrodes by forming boronate ester bonds between sugar residues of HbA1c and PBA moieties on the surface, followed by labeling of the surface with GOx. The amperometric response of the electrodes to glucose decreased with increasing HbA1c levels in the samples because GOx binds only to the HbA1c-free region on the surface. These sensors exhibited linear responses to HbA1c levels of 2.5%–15% [40] and 4.5%–15% [41], both of which cover the range of values required for HbA1c assays used to diagnose diabetes mellitus. However, these sensors are prone to interference from blood sugars and other glycated proteins, thus removal of interfering materials is required to obtain accurate determination of HbA1c levels in the blood.

**Figure 3:**
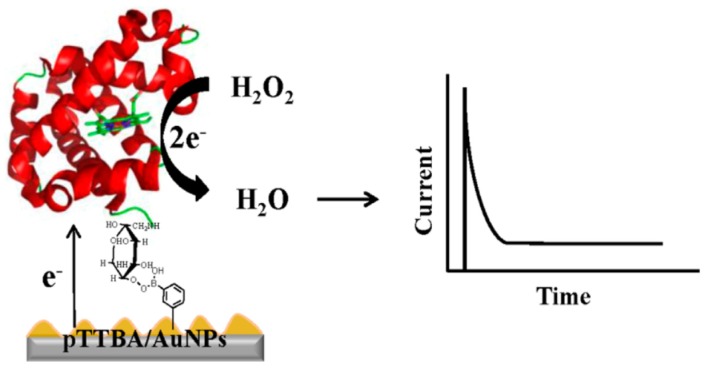
An amperometric HbA1c sensor based on pTTBA/AuNPs-modified electrode. Reprinted with permission from Shim *et al.* [42].

Enzyme labeling is not necessarily required for the construction of amperometric sensors because HbA1c catalyzes the redox reaction of hydrogen peroxide (H_2_O_2_). In fact, disposable amperometric HbA1c sensors have been constructed using a screen-printed electrode modified with PBA-appended poly(thiophene)/Au nanoparticles (pTTBA/AuNPs) [42]. The modified electrode binds HbA1c to the surface through boronate ester bonds. The catalytic current generated through the reduction of H_2_O_2_ by the confined HbA1c was monitored as an analytical signal (Figure 3). Under optimum conditions, the sensor exhibited a linear dynamic range from 0.1% to 1.5% HbA1c. The sensor was also operated in impedometric mode, in which the impedance of the sensor increased with increasing concentrations of HbA1c. The linear dynamic range in this mode was 0.5%–6.0%. Thus, the dynamic range was higher in impedometric mode than in amperometric mode. The disposable sensor was successfully used for analysis of fingerprick blood samples.

The HbA1c sensors reported by Yoon *et al*. [40,41] relied on GOx-catalyzed oxidation of glucose, in which H_2_O_2_ is produced as a reaction product. The pTTBA/AuNPs-based HbA1c sensors acquired output signals in the presence of H_2_O_2_ [42]. Consequently, careful attention was required to detect possible chemical reactions between H_2_O_2_ and boronic acid derivatives, because PBAs are known to be converted to phenol derivatives as a result of oxidative cleavage of a carbon-boron by H_2_O_2_ [43,44,45,46]. Another concern is that glucose can competitively bind to boronic acid residues on the electrode surface to replace HbA1c. In fact, glucose-induced decomposition of PBA assemblies has been reported [47,48,49,50].

Label-free HbA1c sensors can be developed based on PBA derivatives containing redox-active moieties. For instance, voltammetric HbA1c sensors have been constructed using PBA-modified pyrroloquinoline quinone (PBA-PQQ) [51]. The redox signal of the sensor originating from the PBA-PQQ moiety decreased with increasing HbA1c concentrations from 0 to 10% in the samples. Poly(3-aminophenylboronic acid) nanoparticle-coated electrodes have also been used to construct HbA1c sensors [52]. The peak current of the sensor in differential pulse voltammogram decreased with increasing HbA1c concentrations in the range 0.975–156 μM. The sensor showed high selectivity toward HbA1c over glucose, galactose, mannose, serum albumin, ascorbic acid, uric acid, dopamine, and unmodified Hb. Ferrocene (Fc)-modified electrodes were also used as label-free HbA1c sensors. HbA1c sensors were constructed by coating the surface of glassy carbon electrodes with mixed monolayers of oligo(phenylethynylene) molecular wire [53] or Au nanoparticles modified with Fc moieties [54]. The Fc group was modified with a glycosylated penta-peptide as an epitope to which anti-HbA1c antibody could bind. The sensors exhibited redox signals at 0.3–0.4 V in square wave voltammograms, which originated from the Fc moieties. The redox signal decreased upon binding anti-HbA1c to glycosylated penta-peptide on the electrode. In a competitive inhibition assay, the redox signal increased with increasing HbA1c levels in the range of approximatly 4.5%–15% for both sensors. Fc-modified electrodes may be promising for the construction of amperometric and voltammetric sensors because of the reasonable stability and structural versatility of Fc derivatives [55,56].

### 2.2. Potentiometric Sensors

A potentiometric mode in signal transduction is another option in the development of HbA1c sensors. HbA1c sensors were fabricated based on extended-gate ion-sensitive field effect transistors (ISFET) coupled with anti-HbA1c antibody [57,58,59]. The output signals of the ISFET sensor were recorded in differential mode, in which the gate potential of the reference gate was subtracted from that of antibody-immobilized gate to compensate for the effects of non-specific protein adsorption. The use of Au nanoparticles and poly(pyrrole)-Au composites as surface modifiers improved the responses of the ISFET HbA1c sensors.

An interesting strategy for the potentiometric determination of HbA1c was reported, in which alizarin red S (ARS) was used as a redox indicator [60]. The formation of ARS-PBA complex shifted the redox potential of ARS negatively, while the potential shifted positively upon the binding of HbA1c to ARS-PBA, depending on the concentration of HbA1c. Based on this protocol, the percentage of HbA1c in blood hemolysate samples was successfully determined. This protocol would be useful for the determination of HbA1c if the ARS-PBA complex could be confined to the surface of the electrode. ARS-PBA complexes have been used for constructing non-enzymatic sensors for sugars and catechols [61,62,63].

**Figure 4:**
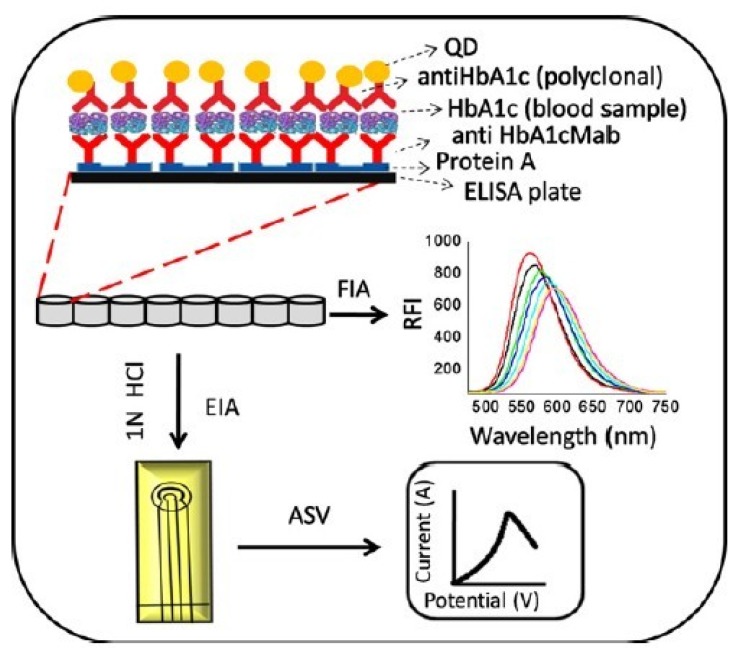
Anodic stripping voltammetry (ASV) and fluorescence immune assay (FIA) HbA1c sensor based on anti-HbA1c/CdTe QDs conjugates. Reprinted with permission from Suri *et al.* [65].

Antibody-modified Au nanoparticles and CdTe quantum dots (QDs) were employed for optical and electrochemical determination of HbA1c. The aggregation of anti-HbA1c-modified Au nanoparticles was suppressed in the presence of HbA1c [64]. The aggregation process was monitored by color changes as well as changes in zeta potential. The zeta potential of the aggregates shifted with increasing concentrations of HbA1c over 1–4 μg/mL. This observation was attributed to the screening of negative charges on the surfaces of the aggregates. However, anti-HbA1-conjugated CdTe QDs were used for a sandwich immunoassay of HbA1c on the basis of fluorometric and electrochemical detection modes [65] (Figure 4). An advantage of this protocol is that HbA1c determination requires only 1 μL blood sample diluted 1:500.

### 2.3. Impedometric Sensors

It is reasonable to assume that changes in impedance may be observed upon specific binding of proteins on the surface of electrodes because of hindered electron transfer. Based on this hypothesis, HbA1c sensors were prepared by depositing PBA-modified graphene oxide on the surface of glassy carbon electrodes [66]. Impedance measurements were carried out in solutions containing 2.5 mM Fe(CN)_6_^3−^/ Fe(CN)_6_^4−^ ions in the presence of HbA1c. The electron transfer resistance of the electrodes linearly increased with increasing concentrations of HbA1c over 2.4–12.0 μM owing to the specific binding of HbA1c to the electrode surfaces through boronate ester formation. In this work, GO was successfully utilized as an electrode modifier to which PBA was covalently attached. The high conductivity and wide surface area of GO contributed to the enhanced performance characteristics of the HbA1c sensors, as is the case for other carbon nanomaterial-based sensors [67,68,69,70].

Parallel electrodes integrated into microfluidic devices as well as ring-shaped interdigitated electrodes have been used for preparing HbA1c sensors. For this purpose, a pair of parallel-facing electrodes, coated with a thiopheneboronic acid monolayer, was fabricated and combined with a polydimethylsiloxane chamber to record the changes in impedance upon HbA1c binding [71]. The impedance of the sensors logarithmically increased with increasing concentrations of HbA1c in the range 10–100 ng/mL. The use of ring-shaped interdigital electrodes enabled determination of HbA1c from 1% to 15% in 200 ng/mL samples [72,73]. Impedance sensors can be operated under label-free and additive-free conditions.

### 2.4. Miscellaneous Sensors

The weight of surface-bound proteins can be determined using quartz crystal microbalance (QCM) [74] and surface plasmon resonance (SPR) spectroscopy [75]. In fact, gravimetric HbA1c sensors based on QCM, in which the weight of HbA1c adsorbed on a quartz resonator was detected as a change in resonance frequency, have been reported. A quartz resonator coated with a thin layer of Au was modified with a phenylboronic acid monolayer to bind HbA1c, and 4%–15% HbA1c in blood could be determined; the total working ranges of HbA1c and Hb were 10–90 and 50–2000 μg/mL, respectively [76]. The use of thicker films composed of cross-linked albumin as a coating was unsuccessful, probably because the access of HbA1c to the binding site was inhibited [77]. Also, an electronic nose system based on QCM sensors has been proposed for determining HbA1c levels through the detection of acetone in exhaled breath [78]. The electronic nose system may be promising as an invasive HBA1c assay if the accuracy of the sensor can be further improved. Recent progress made in the development of the electronic nose for biomedical applications has been comprehensively reviewed [79].

SPR spectroscopy is based on the determination of variations in the refractive index within the environmental medium of a thin Au layer, and is currently used for characterizing protein binding. Chen and coworkers studied binding interactions between a PBA monolayer and HbA1c using this method [80]. The surface of an Au-coated SPR probe was modified with a self-assembled monolayer of PBA for binding HbA1c. The output signal was linearly dependent on the concentration of HbA1c in the range 0.43–3.49 μg/mL.

Recently, lectin-based determination of HbA1c has been studied [81]. HbA1c solutions were mixed with glucose-selective lectin concanavalin A (Con A) to form insoluble aggregates. The amount of HbA1c/Con A aggregates was linearly dependent on the level of HbA1c in the samples; satisfactory correlation with the values obtained by standard HPLC was found. This protocol may be useful for the routine analysis of HbA1c in clinical laboratories because it does not require sophisticated instruments. Various lectin proteins with different sugar selectivity levels are commercially available for bioanalytical research [82,83,84,85]. In this context, highly sensitive biosensors for glycoproteins have been constructed based on lectin-modified Au nanoparticle-coated electrodes [86]. A *Sambucus nigra* agglutinin was used for detecting glycoprotein fetuin, which contains 8.7% sialic acid. The lectin-based sensors exhibited changes in charge transfer resistance in electrochemical impedance spectroscopy at 10^−17^–10^−11^ M fetuin, whereas the response to asialofetuin (0.5% of sialic acid residue) was lower within the same concentration range.

## 3. Sensors for Indirect HbA1c Determination: Fructosyl Valine Sensors

Proteolytic digestion of HbA1c releases fructosyl valine (FV) fragments from the *N*-terminal of β-chains. Therefore, HbA1c can be determined indirectly by detecting FV after digestion of HbA1c. This section focuses on electrochemical FV sensors, which are constructed using an enzyme or molecular imprinted polymer (MIP).

### 3.1. Enzyme Sensors

The pioneering works of Sode and coworkers established that amperometric FV sensors could be constructed by immobilizing fructosyl amine oxidase (FAOx) on the surface of an electrode [87,88,89]. FAOx catalyzes the oxidation reaction of FV to produce H_2_O_2_, which is detected through electro-oxidation on the electrode (Equations (1) and (2)).

(1) FV + H_2_O + O_2_ → valine + glucoson + H_2_O_2_

(2) H_2_O_2_ → O_2_ + 2H^+^ + 2e^−^

FAOx was confined in a film consisting of stilbazole-bearing poly(vinyl alcohol) (PVA-SbQ) through photochemical cross-linking, and immobilized on the surface of a platinum (Pt) electrode. Flow-injection analysis (FIA) equipped with the FAOx-immobilized electrode exhibited responses to FV within the range 0.2–10 mM. The FIA system was used consecutively to determine FV in more than 120 samples within 20 h. The response of the sensor to FV was five times higher than that to N^ɛ^-fructosyl lysine, a digestion fragment released from glycated albumin [88]. The protein engineering of FAOx was recently reviewed [90].

**Figure 5:**
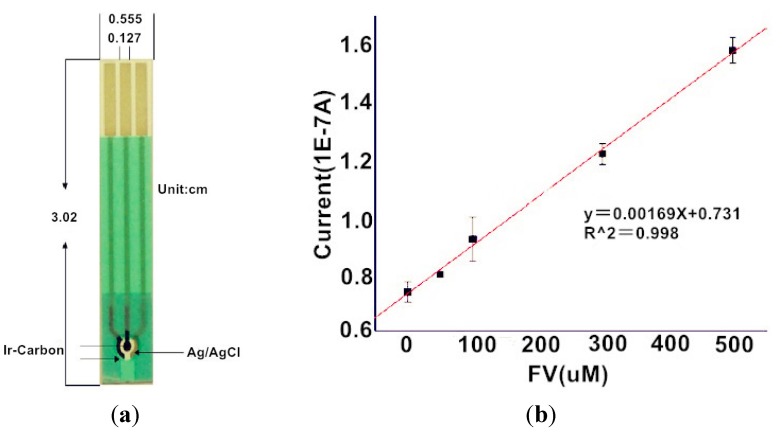
FAOx-modified Ir electrode and a calibration graph of the sensor to FV. Reprinted with permission from Fang *et al.* [91]. (**a**) The configuration of the sensor; (**b**) The calibration graph for the FV sensor.

Nanoparticulate materials are promising tools for improving the performance characteristics of FAOx-based FV sensors. A disposable FV sensor using thick film screen-printed electrodes coupled with FAOx and iridium (Ir) nanoparticles has been reported [91]. Carbon-ink-containing Ir and FAOx was deposited on the surface of the electrode (Figure 5). FV sensors were operated at a relatively low electrode potential (0.25 V *vs.* Ag/AgCl) to detect enzymatic generation of H_2_O_2_. The sensor showed a linear response to FV values of over 0–500 μM (Figure 5). Another study employed FAOx-modified Fe_3_O_4_ and ZnO nanoparticles to construct amperometric FV sensors. The surfaces of Fe_3_O_4_ nanoparticles were coated with chitosan followed by FAOx attachment through glutaraldehyde cross-linking [92]. The sensors exhibited responses to FV levels as low as 0.1 mM. The FAOx sensors were used repeatedly, over 250 times in three months. In addition0, ZnO-based sensors were prepared by consecutive deposition of a poly(pyrrole) layer, ZnO nanoparticles, and FAOx on Au electrodes [93]. The ZnO-based sensors were used for detecting FV levels as low as 0.05 mM, with a linear response range of 0.1–3.0 mM in pH 7.0 medium. The sensors were successfully used to determine HbA1c levels in the digested whole blood of diabetic patients.

### 3.2. MIP-Based Sensors

**Figure 6:**
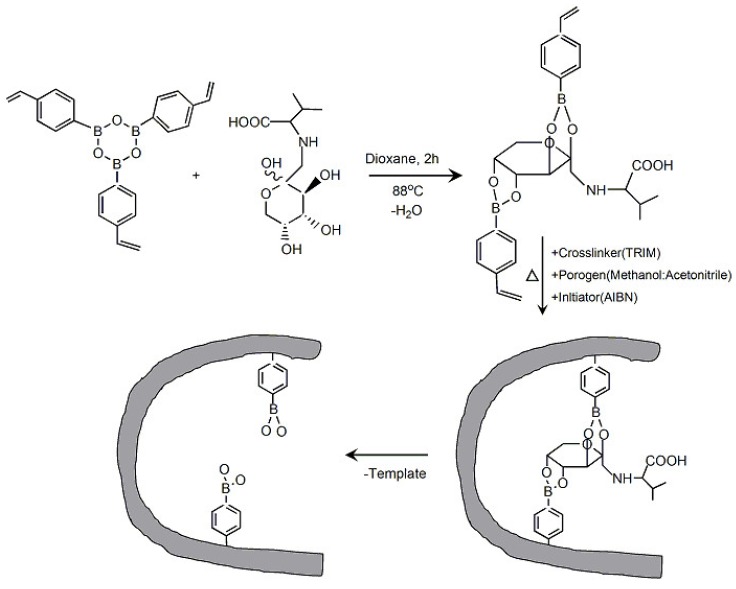
Synthesis of PBA-based MIP for FV sensors. Reprinted with permission from Katterle *et al.* [100].

MIPs have been widely studied with the aim of developing polymeric materials with high affinity to target molecules [94]. In fact, several groups have reported FV-selective MIPs in the construction of FV sensors. It was found that FV is catalytically dehydrogenated by poly(vinyl imidazole) (PVI) in the presence of an electron acceptor [95]. Based on this finding, amperometric FV sensors were prepared using carbon paste electrodes modified with PVI. The sensors exhibited a linear response to FV at concentrations of 0.02–0.7 mM in the presence of 1-methoxyphenazinemethosulphate (m-PMS) as an electron acceptor. PVI chains and PBA residues were used to synthesize catalytically active MIPs, in which PBA bound to FV through boronate ester bonds [96,97,98]. Carbon paste electrodes modified with the MIP showed amperometric responses to 0.2–0.8 mM of FV, while the response to N^ɛ^-fructosyl lysine was lower. Yamazaki recently synthesized soluble MIPs for constructing MIP-modified Au electrodes sensitive to FV [99]. *N*-methacryl-l-histidine, 4-vinylphenylboronic acid, and acrylamide were co-polymerized in the presence of a small amount of cross-linker. The resulting soluble MIP was coated on the surface of an Au electrode to construct an FV sensor. The sensor showed amperometric responses to FV in the concentration range 0.05–0.6 mM. Materials for synthesizing FV-selective MIPs were recently optimized by Katterle and coworkers [100]. They used a 4-vinylphenylboronate ester of FV as a monomeric material for the preparation of an MIP, in which the geometric positions of the PBA residues were suitably arranged for binding FV (Figure 6). MIPs used in the study showed higher selectivity to FV than those prepared using fructose and pinacol imprinting. The same group developed a thermometric MIP sensor for FV by combining thermistor and the MIP [101]. Temperature changes upon FV binding to MIP were observed in the concentration range 0.25–5.0 mM.

A potentiometric FV sensor based on MIPs was reported [102]. The surface of indium-tin oxide (ITO) electrode was coated with poly(aminophenylboronic acid) (APBA) film by *in-situ* polymerization in the presence of FV. The open-circuit potentials of the APBA-coated ITO electrodes were recorded as an output signal of the sensor. The electrode potential of the sensors depended on the concentrations of fructose, glucose, and FV in the sample solutions as a result of selective binding of the compounds, although the response to FV was unstable. Potentiometric FV sensors may be useful because of their simple configuration, particularly if the response to FV could be improved.

### 3.3. Miscellaneous Sensors

Glassy carbon paste electrodes (GCPE), which were prepared using commercially available glassy carbon powder of diameter 20–50 nm, are sensitive to FV [103]. FV was oxidized into an imine derivative at 0.7–0.9 V on the surface of GCPE at pH 7.4. The oxidation current of FV on GCPE was linearly dependent on the concentration of FV within the range 0.1–1.0 mM, suggesting potential use of GCPE for the direct determination of FV. Analogous to the successful use of FcBA in the voltammetric determination of diol compounds, the reduction peak of FcBA in the cyclic voltammogram shifted in the presence of FV [104]. FcBA-based systems may be useful if FcBA could be immobilized on the electrode surface without adding to sample solution.

## 4. Conclusions

Much attention has been devoted to the development of HbA1c sensors for the diagnostic detection of blood glucose levels over the 2–3 months prior to testing. Two different protocols are available for the construction of HBA1c sensors: direct detection of HbA1c and indirect detection of digested products of HbA1c. The former sensors rely on the selective binding of HbA1c onto the surface of electrodes followed by electrical signal transduction in amperometry, voltammetry, potentiometry, or impedometry. Antibodies, lectins, and binding proteins, such as haptoglobin, have been successfully used for the selective binding of HbA1c. Alternatively, glycated amino acid FV produced by the proteolytic digestion of HbA1 is detected by enzyme-modified electrodes or MIP-based sensors. Both types of sensors can successfully determine the levels of HbA1c in diabetic patients. Compared with the temporal data obtained by glucose biosensors, HbA1c sensors are more useful for monitoring the long-term glycemic state of diabetic patients.
